# Association Between Single Nucleotide Polymorphisms in PPARA and EPAS1 Genes and High-Altitude Appetite Loss in Chinese Young Men

**DOI:** 10.3389/fphys.2019.00059

**Published:** 2019-02-04

**Authors:** Wenxu Pan, Chuan Liu, Jihang Zhang, Xubin Gao, Shiyong Yu, Hu Tan, Jie Yu, Dehui Qian, Jiabei Li, Shizhu Bian, Jie Yang, Chen Zhang, Lan Huang, Jun Jin

**Affiliations:** ^1^Department of Cardiology, Xinqiao Hospital, Army Medical University (The Third Military Medical University), Chongqing, China; ^2^Institute of Cardiovascular Diseases, Xinqiao Hospital, Army Medical University (The Third Military Medical University), Chongqing, China

**Keywords:** high altitude, appetite loss, hypoxia, *PPARA*, *EPAS1*, single nucleotide polymorphism

## Abstract

Appetite loss is a common symptom that occurs in high altitude (HA) for lowlanders. Previous studies indicated that hypoxia is the initiating vital factor of HA appetite loss. *PPARA, EPAS1, EGLN1, HIF1A, HIF1AN*, and *NFE2L2* play important roles in hypoxic responses. We aimed to explore the association of these hypoxia-related gene polymorphisms with HA appetite loss. In this study, we enrolled 416 young men who rapidly ascended to Lhasa (3700 m) from Chengdu (<500m) by plane. *PPARA, EPAS1, EGLN1, HIF1A, HIF1AN*, and *NFE2L2* were genotyped by MassARRAY. Appetite scores were measured to identify HA appetite loss. Logistic regression and multiple genetic models were tested to evaluate the association between the single nucleotide polymorphisms (SNPs) and risk of HA appetite loss in crude and adjusted (age and SaO_2_) analysis. Subsequently, Haploview software was used to analyze the linkage disequilibrium (LD), haplotype construction and the association of diverse haplotypes with the risk of HA appetite loss. Our results revealed that allele “A” in *PPARA* rs4253747 was significantly associated with the increased risk of HA appetite loss. Codominant, dominant, recessive, and log-additive models of *PPARA* rs4253747 showed the increased risk of HA appetite loss in the crude and adjusted analysis. However, only dominant, overdominant, and log-additive models of *EPAS1* rs6756667 showed decreased risk of HA appetite loss in the crude and adjusted analysis. Moreover, the results from haplotype-based test showed that the rs7292407-rs6520015 haplotype “AC” was associated with HA appetite loss in the crude analysis rather than the adjusted analysis. In this study, we first established the association of SNPs in *PPARA* (rs4253747) and *EPAS1* (rs6756667) genes with susceptibility to HA appetite loss in Han Chinese young men. These findings provide novel insights into understanding the mechanisms involved in HA appetite loss.

## Introduction

High-altitude (HA) appetite loss is a common symptom of acute mountain sickness (AMS) (Wasse et al., 2012; Matu et al., 2017a) which occurs in lowlanders unacclimated to the environment of hypobaric hypoxia in plateau, and with rapidly (<48 h) ascending to HA (>2500 m), suppression of appetite and energy intake appear (Westerterp-Plantenga, 1999; Matu et al., 2017b). The incidence of HA appetite loss is increasing along with the altitude and approximately 20% at 4243 m (Hackett et al., 1976). With acclimation, the symptoms of AMS gradually disappeared, HA appetite loss in some patients still exists for a long time (Tschöp and Morrison, 2001). Although HA appetite loss is not a fatal disease, severe HA appetite loss can cause weight loss, seriously impair physical health and reduce work capacity, and even compel subjects to return to low land (Hoppeler and Vogt, 2001). However, so far, effective prediction or prevention for HA appetite loss is lacking. Therefore, HA appetite loss is an important unresolved topic.

The mechanisms of HA appetite loss are multifactorial, and they include physiological, environmental and genetic factors. Alterations of appetite-regulating peptides following HA exposure have been considered as a potential mechanism of HA appetite loss for many years (Camilleri, 2015; Debevec, 2017). These peptides include orexigenic hormone (e.g., ghrelin) and anorexigenic hormones (e.g., leptin, glucagon-like peptide-1, peptide YY, and cholecystokinin) (Marić et al., 2014; Debevec, 2017). Through circulation or vagal nerve via the nucleus of the solitary tract, these peptides conveyed signs of satiation/hunger to the nerve center (e.g., hypothalamic, brain), leading to an increase or decrease of food intake (Camilleri, 2015; Ueno and Nakazato, 2016). Leptin is a hypoxia-sensitive gene, previous study showed that hypoxia increased the expression and secretion of leptin (Wang et al., 2008). Furthermore, elevated leptin concentrations at HA were found to be associated with loss of appetite, which indicated that leptin may play a critical role in HA appetite loss (Tschöp et al., 1998). Additionally, HA hypoxia induces oxidative stress and subsequently promotes expression of some inflammatory cytokines, such as interleukin-1, interleukin-6, interleukin-8, and tumor necrosis factor-alpha, which may suppress appetite via activation of the hypothalamic appestat (Plata-Salamán, 2001). Evidence has shown that the activation of hypoxia-inducible genes which may regulate the above mentioned factors is the initial step following HA exposure and subsequent HA appetite loss (Yang et al., 2017; Murray et al., 2018). However, among the hypoxia-inducible genes, it is still unclear which of them are associated with HA appetite loss.

It is well known that the genes *peroxisome proliferator activated receptor alpha* (*PPARA*, encoding Peroxisome proliferator-activated receptor α), *endothelial PAS domain protein 1* (*EPAS1*, encoding hypoxia inducible factor-2α), *egl-9 family hypoxia inducible factor 1* (*EGLN1*, encoding prolyl hydroxylase domain-containing proteins-2), *hypoxia inducible factor 1 alpha subunit* (*HIF1A*, encoding hypoxia inducible factor 1-α), *hypoxia inducible factor 1 alpha subunit inhibitor* (*HIF1AN*, encoding inhibitor for hypoxia inducible factor 1-α), and *nuclear factor erythroid 2-like 2* (*NFE2L2*, encoding nuclear factor E2-related factor-2) are involved in hypoxia adaptation through encoding transcription factors, and they play important roles in the hypoxia pathway and oxidative stress (Simonson et al., 2010; Zhang et al., 2014; Son et al., 2017). It was reported that polymorphisms of the *PPARA* gene were associated with many diseases, such as liver disease (Li et al., 2014; Kersten and Stienstra, 2017), celiac disease (Mostowy et al., 2016), and hyperlipidemia (Eurlings et al., 2002). And numerous studies have demonstrated that the polymorphism of *EPAS1* is related to pancreatic cancer (Zhang et al., 2017) and inflammatory bowel disease (Xue et al., 2013). Additionally, previous studies showed that *EGLN1, HIF1A, HIF1AN*, and *NFE2L2* were associated with metabolic syndrome, digestive tract cancer, type 2 diabetes, alcoholic liver disease and chronic gastritis (Arisawa et al., 2007; Zhai et al., 2012; Matsuura et al., 2013; Sun et al., 2015; Xu et al., 2016). Furthermore, these hypoxia-inducible genes are also involved in the expression of appetite regulating peptides and oxidative stress inflammatory cytokines (Heun et al., 2012; Xue et al., 2013; Rahtu-Korpela et al., 2016; Gladek et al., 2017; Horscroft et al., 2017; Son et al., 2017). However, whether the polymorphisms of these hypoxia-inducible genes are associated with the incidence of HA appetite loss needs to be investigated.

Based on these considerations, we aimed to explore the relationships between the possible SNPs of the above-mentioned hypoxia-inducible genes such as *PPARA, EPAS1, EGLN1, HIF1A, HIF1AN*, and *NFE2L2* with the occurrence of HA appetite loss under acute HA exposure in Han Chinese young men. Duo to the well known roles of the appetite-regulating peptides, these genes (such as that of leptin) were not selected in our study. Additionally, those Tibetan-specific SNPs (such as EGLN1- D4E variants), Denisovan SNPs/non-Denisovan Tibetan selected SNPs and *PPARA* Tibetan selected SNPs (Simonson et al., 2010; Huerta-Sánchez et al., 2014; Hu et al., 2017) are remarkably enriched in highlanders (e.g., Tibetans), which have been proved to be associated with HA adaption were also not selected.

## Materials and Methods

### Study Participants

To eliminate the confounding effect of gender, 416 healthy Han Chinese young men aged from 18 to 45 years old were recruited in June and July of 2012. Subjects who matched any conditions as follows were excluded from this research: participants who were exposed to HA (2500 m) in the recent 6 months or had taken preventive measures (e.g., acetazolamide, antipyretic analgesics or steroids) or were diagnosed with cardiovascular diseases, respiratory diseases, gastrointestinal diseases, anorexia, neurological diseases, mental disease, malignant tumors, liver, or kidney disorders. In addition, subjects with poor compliance were excluded too. One week before ascending to HA, venous blood samples were collected from all subjects for SNP analysis. Subjects enrolled in this study took a trip from Chengdu (approximately 500 m above sea level, asl) to Lhasa (approximately 3700 m, asl) by airplane, 48–72 h after arrival, the physiologic parameters of oxygen saturation (SaO2) were examined by Pulse Oximeter (NONIN-9550, Nonin Onyx, Plymouth, MN, United States). Our study was approved by the Ethics Committee of Xinqiao Hospital, Third Military Medical University (identification code: 2012014 approved on 9 May 2012). All participants were informed with the research contents and signed informed consents.

### Appetite Scoring

Before ascending to HA, all of the subjects were free of any gastrointestinal symptoms, including loss of appetite, nausea, and vomiting. The present appetite evaluation was based on the validated six-point Likert scale with some modifications, and the score was rated as 0 = no, 1 = litter, 2 = moderate, 3 = moderate to severe, 4 = severe, and 5 = very much severe (Valentova et al., 2016). Before completing the questionnaire, subjects were informed with appetite perceptions which contained of the following subjective feelings (hungry, fullness, satiety and prospective food consumption). 48–72 h after arrival HA, subjects were asked to answer the questionnaire “How would you rate the severity of appetite loss?”. Based on the subjects’ general feelings of appetite perceptions, the score was rated from 0 (no) to 5 (very much) by Likert scale. According to previous study, a cut-off value ≥ 1 on the scale was used to define appetite loss and then the subjects were divided into the control group (0, without appetite loss) and case group (≧1, with appetite loss) (Saitoh et al., 2017). These processes also have been described in Supplementary Material online.

### SNP Selection and Genotyping

As mentioned above, 27 putative functional SNPs in PPARA, EPAS1, EGLN1, HIF1A, HIF1AN, and NFE2L2 were selected from the dbSNP Database^1^ and all MAF values ≧ 0.05. We employed Sequenom^®^ Assay Design software (version 3.1, Sequenom Inc, San Diego, CA, United States) to design PCR primer pairs, which were synthetized by Sangon Biotech (Shanghai, China). Genomic DNA was extracted from blood samples by using the standard protocols of the Relax Gene Blood DNA System (TIANGEN, Beijing, China). The DNA samples were stored at -20°C until used. The genotyping was performed by MassARRAY^®^ MALDI-TOF System (Sequenom Inc., San Diego, CA, United States), and the approaches were described in detail in our previous study (Zhang et al., 2014). To guarantee the quality of results, we added a blank control and duplicate sample detection in the event of ambiguous results.

### Statistical Analysis

The statistical analysis was implemented by SPSS 19.0 (SPSS Inc., Chicago, IL, United States) and SNPstats (Solé et al., 2006). Independent sample T-tests were performed to compare the means of data, which were in accordance with normal distribution, and the results were expressed as the means ± SD (standard deviation). Chi squared test was applied to confirm deviation from Hardy-Weinberg equilibrium (HWE) and allele frequencies. Odd ratios (OR) and 95% confidence intervals (CI) were performed by unconditional logistic regression, adjusted for age and SaO2. Finally, the Haploview 4.2 (Barrett et al., 2005) software was employed to analyze the LD and haplotype construction, and further analyze the risk association between haplotypes and HA appetite loss. The HWE was agreed by *P* > 0.05, else difference of *P* < 0.05 was statistically significant.

## Results

In this study, we enrolled 416 participants in total, including 106 subjects with HA appetite loss (case), and 310 subjects without HA appetite loss (control). The incidence of HA appetite loss was 25.48%. As shown in Table 1, there were no significant differences in age, height, weight, body mass index (BMI), and SaO_2_ between the case and control groups.

**Table 1:** Characteristics of the subjects.

Variable	Case (*n* = 106)	Control (*n* = 310)	*P value*
Age (year)	23.15 ± 3.98	22.82 ± 3.57	0.629
Height (cm)	171.50 ± 4.79	171.80 ± 4.68	0.660
Weight (kg)	64.70 ± 7.81	64.47 ± 7.38	0.494
BMI (kg/cm^2^)	21.68 ± 2.09	21.83 ± 2.23	0.544
SaO_2_ (%)	87.97 ± 3.09	88.30 ± 2.89	0.308


*P < 0.05 indicates statistical significance; BMI, body mass index; SaO_2_, oxygen saturation; Case, with appetite loss; Control, without appetite loss.*

From Table 2, we obtained the basic information about the SNPs regarding our study, which contained gene, allele, minor allele frequency (MAF), and Hardy-Weinberg equilibrium (HWE) test results. The results of all the HWE tests in our study were >0.05. After the comparison of allele frequency distributions in cases and controls, we found that only one SNP (rs4253747, *PPARA*) was significantly associated with HA appetite loss (A vs. T: *p* = 0.022, odds ratio [OR] = 1.79, 95% confidence interval [CI] = 1.08–2.95).

**Table 2:** Allele frequencies in cases and controls and odds ratio estimates for HA appetite loss risk.

SNP	Gene	Allele	MAF- case	MAF- control	*P* value for HWE test	OR (95%CI)	*P* value
rs135538	*PPARA*	C/G	0.490	0.424	0.13	1.31 (0.8–2.04)	0.233
rs4253623	*PPARA*	G/A	0.146	0.126	0.80	1.22 (0.65–2.28)	0.539
rs4253681	*PPARA*	C/T	0.247	0.192	0.27	1.38 (0.81–2.33)	0.231
rs4253747	*PPARA*	A/T	0.297	0.192	0.27	1.79 (1.08–2.95)	0.022^∗^
rs6520015	*PPARA*	C/T	0.133	0.197	0.28	0.63 (0.33–1.17)	0.141
rs7292407	*PPARA*	A/C	0.104	0.173	0.10	0.60 (0.30–1.21)	0.150
rs13419896	*EPAS1*	A/G	0.278	0.321	0.70	0.82 (0.51–1.34)	0.429
rs1868092	*EPAS1*	A/G	0.066	0.085	0.26	0.74 (0.31–1.76)	0.500
rs4953354	*EPAS1*	G/A	0.122	0.102	1.00	1.56 (0.74–3.28)	0.236
rs6756667	*EPAS1*	A/G	0.080	0.135	0.33	0.59 (0.28–1.25)	0.162
rs2275279	*EGLN1*	T/A	0.240	0.273	0.15	0.85 (0.51–1.41)	0.529
rs2790882	*EGLN1*	G/A	0.442	0.425	0.48	1.07 (0.69–1.68)	0.762
rs480902	*EGLN1*	T/C	0.438	0.421	0.49	1.07 (0.69–1.67)	0.762
rs1339891	*EGLN1*	A/G	0.099	0.089	1.00	1.15 (0.55–2.41)	0.702
rs2066140	*EGLN1*	C/G	0.433	0.421	0.48	1.06 (0.68–1.65)	0.812
rs2486736	*EGLN1*	C/G	0.442	0.420	0.48	1.09 (0.70–1.71)	0.693
rs12434438	*HIF1A*	G/A	0.269	0.243	0.88	1.15 (0.69–1.90)	0.600
rs2301104	*HIF1A*	C/G	0.052	0.079	0.71	0.69 (0.27–1.72)	0.420
rs2301112	*HIF1A*	C/A	0.035	0.040	1.00	0.98 (0.31–3.12)	0.976
rs966824	*HIF1A*	T/C	0.200	0.171	0.84	1.21 (0.69–2.13)	0.504
rs10883512	*HIF1AN*	G/A	0.084	0.074	0.23	1.15 (0.52–2.58)	0.727
rs11190602	*HIF1AN*	C/T	0.142	0.117	0.58	1.26 (0.66–2.42)	0.478
rs2295778	*HIF1AN*	G/C	0.235	0.256	1.00	0.90 (0.53–1.50)	0.673
rs10497511	*NFE2L2*	C/T	0.325	0.302	0.50	1.12 (0.70–1.80)	0.631
rs1962142	*NFE2L2*	T/C	0.278	0.273	1.00	1.04 (0.64–1.69)	0.888
rs2364722	*NFE2L2*	G/A	0.451	0.455	0.73	0.99 (0.63–1.55)	0.959
rs6721961	*NFE2L2*	T/G	0.349	0.321	0.90	1.13 (0.71–1.80)	0.601


*OR, odds ratio; 95%CI, 95% confidence interval; ^∗^P < 0.05 indicates statistical significance; MAF, minor allele frequency; HWE, Hardy-Weinberg equilibrium; SNP, single nucleotide polymorphism; Case, with appetite loss; Control, without appetite loss.*

After being analyzed by five genetic models (codominant, dominant, recessive, overdominant, and log-additive models) for all SNPs and the association with HA appetite loss, the consequences demonstrated that two SNPs (rs4253747, *PPARA* and rs6756667, *EPAS1*) were significantly associated with HA appetite loss in the crude analysis. As Table 3 shows, the codominant model analysis of rs4253747 showed that the genotypes, “AA” (OR = 4.69; 95%CI = 1.77–12.48; *P* = 0.004), were associated with the increased risk of HA appetite loss. From the dominant model, we found that genotype “AT + AA” (OR = 1.79; 95%CI = 1.15–2.80; *P* = 0.010) 0f rs4253747 was associated with the increased risk of HA appetite loss. Under the recessive model, the genotype, “AA” (OR = 3.93; 95%CI = 1.51–10.25; *P* = 0.006) of rs4253747, was related to the increased risk of HA appetite loss. Meanwhile, in the log-additive model, rs4253747 was associated with the increased risk of HA appetite loss (OR = 1.83; 95%CI = 1.26–2.64; *P* = 0.002). In addition, after adjustment for age and SaO_2_, these associations also remained significant (*p* < 0.05).

**Table 3:** Single nucleotide polymorphism of rs4253747 analysis under different genetic models with the risk of HA appetite loss.

SNP	Model	Geno type	Control	Case	Crude		Adjusted^a^	
					
					OR (95%CI)	*P-Value*	OR (95%CI)	*P-Value*
PPARA- rs4253747	Codominant	T/T	199 (64.2%)	53 (50%)	1.00	–	1.00	–
		A/T	103 (33.2%)	43 (40.6%)	1.57 (0.98–2.50)	–	1.59 (1.00–2.54)	–
		A/A	8 (2.6%)	10 (9.4%)	4.69 (1.77–12.48)	0.004^∗^	5.05 (1.88–13.57)	0.002^∗^
	Dominant	T/T	199 (64.2%)	53 (50%)	1.00	–	1.00	–
		A/T-A/A	111 (35.8%)	53 (50%)	1.79 (1.15–2.80)	0.010^∗^	1.83 (1.17–2.86)	0.008^∗^
	Recessive	T/T-A/T	302 (97.4%)	96 (90.6%)	1.00	–	1.00	–
		A/A	8 (2.6%)	10 (9.4%)	3.93 (1.51–10.25)	0.006^∗^	4.19 (1.59–11.02)	0.004^∗^
	Overdominant	T/T-A/A	207 (66.8%)	63 (59.4%)	1.00	–	1.00	–
		A/T	103 (33.2%)	43 (40.6%)	1.37 (0.87–2.16)	0.170	1.38 (0.88–2.18)	0.160
	Log-additive	–	–	–	1.83 (1.26–2.64)	0.002^∗^	1.87 (1.29–2.71)	0.001^∗^


*OR, odds ratio; 95%CI, 95% confidence interval; ^∗^P < 0.05 indicate statistical significance. ^a^Adjusted for age and SaO_2_ in a logistical regression model. SNP, single nucleotide polymorphism; Case, with appetite loss; Control, without appetite loss.*

Furthermore, as shown in Table 4, results from the dominant model analysis showed that the genotype, “AG + AA” (OR = 0.54; 95%CI = 0.30–0.96; *P* = 0.030) of rs6756667, was associated with the decreased risk of HA appetite loss. Under the overdominant model, the genotype, “AG” (OR = 0.57; 95%CI = 0.32–1.01; *P* = 0.047) of rs6756667, was also associated with the decreased risk of HA appetite loss. In addition, in the log-additive model, rs6756667 was significantly related to the decreased risk of HA appetite loss (OR = 0.53; 95%CI = 0.30–0.94; *P* = 0.022). What’s more, after adjustment for age and SaO_2_, these associations also remained significant (*p* < 0.05).

**Table 4:** Single nucleotide polymorphism of rs6756667 analysis under different genetic models with the risk of HA appetite loss.

SNP	Model	Geno type	Control	Case	Crude		Adjusted^a^	
					
					OR (95%CI)	*P-Value*	OR (95%CI)	*P-Value*
EPAS1- rs6756667	Codominant	G/G	229 (73.9%)	89 (84%)	1.00	–	1.00	–
		A/G	78 (25.2%)	17 (16%)	0.56 (0.31–1.00)	0.052	0.56 (0.31–1.00)	0.053
		A/A	3 (1%)	0 (0%)	0.00	–	0.00	–
	Dominant	G/G	229 (73.9%)	89 (84%)	1.00	–	1.00	–
		A/G-A/A	81 (26.1%)	17 (16%)	0.54 (0.30–0.96)	0.030^∗^	0.54 (0.30–0.96)	0.030^∗^
	Recessive	G/G-A/G	307 (99%)	106 (100%)	1.00	–	1.00	–
		A/A	3 (1%)	0 (0%)	0.00	0.180	0.00	0.180
	Overdominant	G/G-A/A	232 (74.8%)	89 (84%)	1.00	–	1.00	–
		A/G	78 (25.2%)	17 (16%)	0.57 (0.32–1.01)	0.047^∗^	0.57 (0.32–1.02)	0.048^∗^
	Log-additive	–	–	–	0.53 (0.30–0.94)	0.022^∗^	0.54 (0.30–0.94)	0.023^∗^


*OR, odds ratio; 95%CI, 95% confidence interval; ^∗^P < 0.05 indicate statistical significance. ^a^Adjusted for age and SaO_2_ in a logistical regression model. SNP, single nucleotide polymorphism; Case, with appetite loss; Control, without appetite loss.*

**FIGURE 1:**
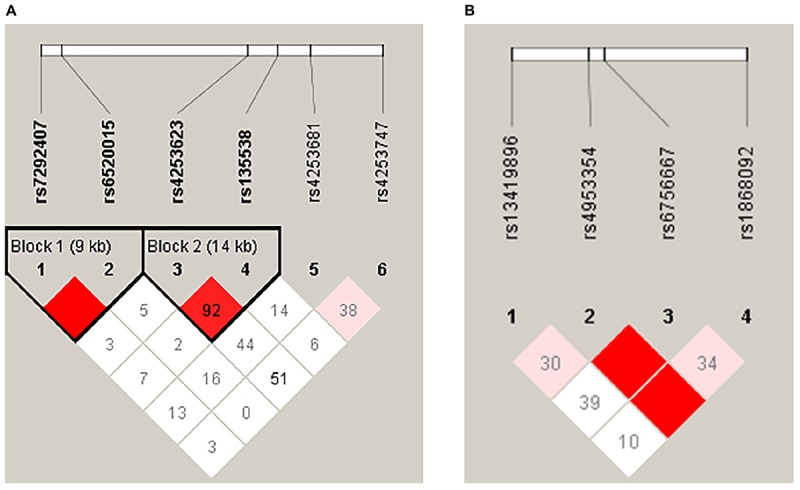
Haplotype block map for SNPs of the *PPARA* **(A)** and *EPAS1* **(B)** genes.

Two LD blocks in the *PPARA* gene (Figure 1A) were found in our results. Block 1 contained rs7292407 and rs6520015, and haplotype “AC” in block 1 was significantly associated with the decreased risk of HA appetite loss in the crude analysis (OR = 0.61; 95%CI = 0.38–0.98; *P* = 0.041); however, no significant association was found after adjustment for age and SaO_2_. Block 2 contained rs4253623 and rs135538, but no significant association was observed for both analyses (Table 5). Moreover, no haplotype block was observed in *EPAS1* SNPs (Figure 1B).

**Table 5:** *PPARA* haplotype frequencies and the association with risk of HA appetite loss.

Block	Haplotype	Freq (case)	Freq (control)	*P^a^*	Crude		Adjusted^b^	
					
					OR (95%CI)	*P*	OR (95%CI)	*P*
1	CT	0.868	0.803	0.035^∗^	1.00	–	1.00	–
	AC	0.112	0.173	0.035^∗^	0.61 (0.38 –0.98)	0.041^∗^	0.62 (0.39 –1.00)	0.050
	CC	0.020	0.024	0.764	0.77 (0.25–2.41)	0.660	0.78 (0.25–2.47)	0.680
2	AG	0.503	0.572	0.080	1.00	–	1.00	–
	AC	0.351	0.302	0.186	1.30 (0.93 –1.82)	0.120	1.32 (0.95 –1.86)	0.100
	GC	0.139	0.121	0.485	1.29 (0.79 –2.11)	0.310	1.34 (0.82 –2.20)	0.250


*^a^Two-sided _X_^2^ test/Fisher’s exact tests. ^b^Adjusted for age and SaO_2_ in a logistic regression model. ^∗^P < 0.05 indicates statistical significance. Block 1, contained rs7292407 and rs6520015 of PPARA; Block 2, contained rs4253623 and rs135538 of PPARA; Case, with appetite loss; Control, without appetite loss.*

## Disscussion

In this study, we first established the association of SNPs in *PPARA* (rs4253747) and *EPAS1* (rs6756667) genes with the susceptibility to HA appetite loss. Our results revealed that the allele “A” in *PPARA* rs4253747 was significantly associated with the increased risk of HA appetite loss whereas the SNPs in *EPAS1* rs6756667 were significantly associated with the decreased risk of HA appetite loss. Moreover, the results from the haplotype-based test showed that the rs7292407-rs6520015 haplotype was associated with HA appetite loss in the crude other than the adjusted analysis.

The *PPARA* gene, which was mapped to chromosome 22q13-31, consists of eight exons (93161 bp transcript, 36997 bp coding sequence) and large introns (containing 56164 bp). *PPARA* gene encoded peroxisome proliferator-activated receptor alpha (PPARα), which is a member of the nuclear receptor transcription factors family and regulates the expression of various target genes in the nucleus, involved in energy homeostasis. Recent studies have confirmed that PPARα is widely expressed in various organs and tissues, such as liver, kidney, heart, small intestine, and brown adipose (Murray et al., 2018). PPARα is the key regulator of glucose and lipid metabolism, immune response, cell differentiation and other physiological processes. It had been reported that PPARα was a protective factor in the pathogenesis of diabetes mellitus (Jay and Ren, 2007). In addition, PPARα variants were associated with hyperlipidemia (Uauy et al., 2000). Moreover, acute HA exposure increased the expression of PPARα, which in turn stimulated gluconeogenesis in the liver, increased circulation blood glucose concentration, and indirectly promoted insulin secretion, the latter perhaps ultimately leads to HA appetite loss (Duraisamy et al., 2015; Horscroft et al., 2017). Additionally, it had been demonstrated that oleoylethanolamide generated from the small intestine exerted the property of appetite inhibition through activation of PPARα (Sarro-Ramírez et al., 2013). Our results illustrated that *PPARA* rs4253747 showed significant association with HA appetite loss. In addition, the variant contributes to the increased risk of HA appetite loss. Furthermore, haplotype analysis showed that rs7292407 and rs6520015 exhibited a strong LD and formed one haplotype block. In addition, the haplotype was associated with the decreased risk of HA appetite loss in the crude analysis. It is well known that *PPARA* rs4253747 is located in the intron region, the specific function of rs4253747 is not clear recently, it may play a role of binding to various transcription factors (e.g., promoter or enhancer). According to our research, further studies may reveal its possible role in regulating HA appetite loss.

The *EPAS1* gene, which is located on chromosome 2 (46297402 bp-46386703 bp), consists of sixteen exons and small introns (which contain 2553 bp), and encode endothelial PAS domain protein 1 (EPAS1), also known as hypoxia inducible factor 2α (HIF-2α), which contains a basic-helix-loop-helix domain, protein dimerization domain, as well as a domain found in proteins in signal transduction pathways that respond to oxygen levels, and plays a crucial role in iron metabolism, red blood cell formation, vascular growth, hypoxia adaptation, fetal lung maturation, liver growth, and other physiological aspects (Tian et al., 1997). *EPAS1* gene is strongly associated with high-altitude illness and was confirmed to be related to high-altitude adaptation in Tibetans. It was reported that *EPAS1* variants were associated with hemoglobin concentration, pulmonary arterial pressure, and nitric oxide production, which acted as an adaptive strategy in response to HA hypoxia (Peng et al., 2017). Additionally, *EPAS1* gene was involved in adipogenesis, and mediated leptin expression (Shimba et al., 2004). It is well known that leptin inhibited appetite via activating pro-opiomelanocortin neurons and depressing neuropeptide Y/agouti-related regulatory peptide AgRP neurons in the arcuate nucleus (Pinto et al., 2004). Therefore, EPAS1 may be involved in HA appetite loss through regulating leptin expression. In our study, we found that *EPAS1* rs6756667 is associated with HA appetite loss. However, the SNP of rs6756667 is also an intronic mutant, its specific function in *EPAS1* gene and the mechanism for reducing risk of HA appetite loss is unclear recently, perhaps the variant of rs6756667 is in LD with true functional variation and/or directly influence the transcription of EPAS1 and is involved in HA appetite loss susceptibility, but the exact mechanism needs further investigation.

Several limitations in our study should be taken into consideration. First, subjects in our study were all restricted to Han Chinese young men, but whether the results could apply to other populations is still unknown. Second, the identification of HA appetite loss was based on a self-report without immediate medical control, which might lead to possible classification bias. The same well-trained operators who adopted strict reading criteria and were blind to the subject’s grouping information performed all examinations. Moreover, the subjects were well-informed to understand and report appetite-related changes accurately. In addition, participants completed the questionnaire in isolation so that social influence did not affect the selection or quality of evaluation. Accordingly, the classification bias would be minimized to the utmost. Third, we only analyzed the association between genes and HA appetite loss; the specific function of these SNPs is still unknown, further study is required to evaluate the function of these SNPs on genes and the mechanism for the functional characterization and biological effects of these gene polymorphisms on appetite. Fourth, due to the limitations of our field study, only the parts of SNPs which may be associated with HA appetite loss are selected. Further study is warranted to identify other possible SNPs and genes which have been proved to play an important roles in HA adaptation or appetite regulation, such as EGLN1-C127S, EGLN1-D4E, c.[12C > G; 380G > C] PHD2 variant and *PKLR* gene (Yi et al., 2010; Xiang et al., 2013).

## Conclusion

This study was the first to investigate the association between the SNPs of *PPARA*, *EPAS1*, *EGLN1*, *HIF1A*, *HIF1AN*, and *NFE2L2* and HA appetite loss in Han Chinese young men. Polymorphisms of *PPARA* rs4253747 increased the risk of HA appetite loss. In contrast, *EPAS1* rs6756667 played a protective role in HA appetite loss. In addition, carriers of the “AC” haplotype of *PPARA* rs7292407-rs6520015 also exhibit a protective role for HA appetite loss. Taken together, our findings may provoke further investigation for exploring the mechanism of HA appetite loss with hypoxia-inducible genes. Moreover, our study may provide novel strategies for prediction, prevention and treatment of HA appetite loss in future, by which the physical health and work capacity of subjects who have to ascend to HA will be effectively preserved.

## Author Contributions

JZ, XG, JJ, and LH conceived and designed the study. WP, CL, SY, SB, HT, JYu, DQ, JL, JYa, and CZ performed the experiments and analyzed the data. WP and CL wrote the paper. JJ and LH critically reviewed the manuscript. All authors approved the final manuscript.

## Conflict of Interest Statement

The authors declare that the research was conducted in the absence of any commercial or financial relationships that could be construed as a potential conflict of interest.

## Footnotes

1 http://www.ncbi.nlm.nih.gov/projects/SNP/
