# Supplementary figures and images for: Association Between Single Nucleotide Polymorphisms in PPARA and EPAS1 Genes and High-Altitude Appetite Loss in Chinese Young Men
Source: Front Physiol. 2019 Feb 4;10:59. doi: 10.3389/fphys.2019.00059 (PMC6369186; doi:10.3389/fphys.2019.00059)

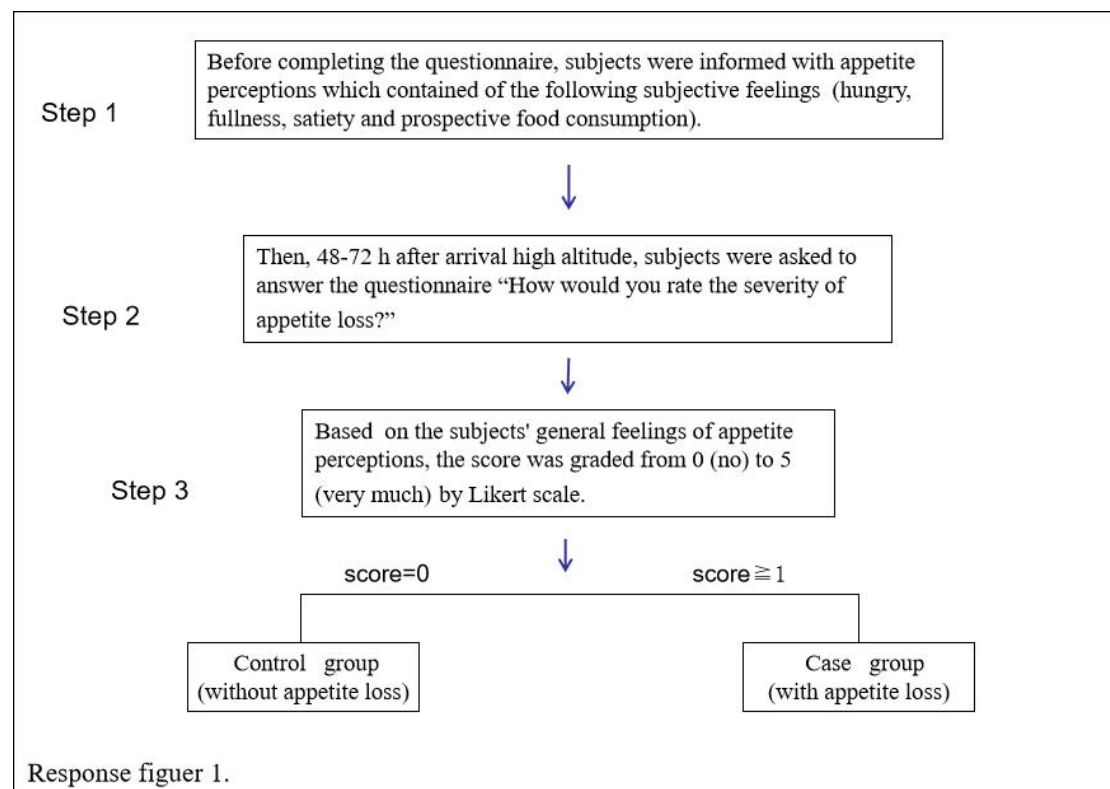

Supplement: Supplementary file 1 [file Data_Sheet_1.PDF]
